# The performance of the Congruence Among Distance Matrices (CADM) test in phylogenetic analysis

**DOI:** 10.1186/1471-2148-11-64

**Published:** 2011-03-09

**Authors:** Véronique Campbell, Pierre Legendre, François-Joseph Lapointe

**Affiliations:** 1 Département de Sciences biologiques, Université de Montréal, C.P. 6128, Succ. Centre-ville, Montréal, Québec, H3C 3J7, Canada

## Abstract

**Background:**

CADM is a statistical test used to estimate the level of Congruence Among Distance Matrices. It has been shown in previous studies to have a correct rate of type I error and good power when applied to dissimilarity matrices and to ultrametric distance matrices. Contrary to most other tests of incongruence used in phylogenetic analysis, the null hypothesis of the CADM test assumes complete incongruence of the phylogenetic trees instead of congruence. In this study, we performed computer simulations to assess the type I error rate and power of the test. It was applied to additive distance matrices representing phylogenies and to genetic distance matrices obtained from nucleotide sequences of different lengths that were simulated on randomly generated trees of varying sizes, and under different evolutionary conditions.

**Results:**

Our results showed that the test has an accurate type I error rate and good power. As expected, power increased with the number of objects (i.e., taxa), the number of partially or completely congruent matrices and the level of congruence among distance matrices.

**Conclusions:**

Based on our results, we suggest that CADM is an excellent candidate to test for congruence and, when present, to estimate its level in phylogenomic studies where numerous genes are analysed simultaneously.

## Background

In phylogenetic studies, data matrices are assembled and analysed to infer evolutionary relationships among species or higher taxa. Depending on the study, character-state data or distance matrices may be used, and several different types of data may be available to estimate the phylogeny of a particular group [1]. An increasing number of phylogenomic studies are published for data sets including more than 100 genes [2-10]. Whereas character-state data (e.g., nucleotide sequences) are commonly used for parsimony, maximum likelihood or Bayesian analyses, distance methods can be selected as an alternative option to decrease computing time when analysing large data sets, or else, can be used in comparative studies where the primary data are not available.

Different approaches have been proposed to analyse the growing amount of information that may originate from different sources. The total evidence approach [11], also called character congruence approach [*sensu *[12]] or combined analysis [*sensu *[13]], combines different data sets in a single supermatrix [14-17]. The taxonomic congruence approach [*sensu *[12]], or consensus approach [13], analyses each matrix separately, and combines the resulting trees *a posteriori *using a consensus [18-22] or a supertree method [23-26]. The pros and cons of these competing approaches have been debated at length in the literature [7,17,21,22,27-32]. An intermediate approach, referred to as the conditional data combination, consists in testing *a priori *the level of congruence of different data sets. Only the data sets that are considered statistically congruent, i.e. in phylogenetic agreement, are combined in a supermatrix. The remaining incongruent data sets are analysed separately [13,19,33-35].

The approach used often depends on the level of congruence or incongruence in the data. In phylogenetic analysis, "incongruence" can be defined as differences in phylogenetic trees. It is observed when different partitions, or data sets, sampled on the same taxa suggest different evolutionary histories [36]. However, incongruence may also arise when the data violate the assumptions of the phylogenetic method. Incongruence among data sets is fairly common and can be present at varying degrees [37]. Hence, statistical tests have been designed to detect the presence of incongruence and its magnitude [36]. In general, such incongruence tests are used to determine if the topological differences observed could have simply arose by chance [38]. The null hypothesis of most of these tests (H_0_) is congruence, i.e., topologically identical trees, where any topological difference is the result of stochastic variation in the data sets [see [22], [38] for reviews]. The most commonly used test of this type is the Incongruence Length Difference test [ILD: [39]]. However, numerous problems are known to be associated to it. For example, type I error rates were shown to be well above the nominal significance level when data sets (with great differences in substitution rates among sites) were compared [40,41]. Therefore, nominal significance levels of 0.01 or 0.001 have been suggested as more appropriate [36]. Also, power was low when short nucleotide sequences simulated on different tree structures were compared [41].

Numerous factors have been described to explain differences in phylogenetic trees obtained from the analysis of data sets containing the same species. A wide range of evolutionary processes may cause nucleotides at different sites to evolve differently, for examples due to their codon positions or to different functional constraints [42-44]. Also, various parts of the genome may have experienced different phylogenetic histories (e.g., mitochondrial vs. nuclear genes) and trees inferred from different data types (e.g., morphological or molecular data) may support different phylogenies [45]. Other evolutionary processes can explain incongruence between data sets: horizontal transfer, duplications, insertions or losses, incomplete lineage sorting, mobile elements, recombination, hybridization and introgression [see [37], [38] for an exhaustive list]. Furthermore, the use of an inappropriate method to analyse a given data set may lead to a spurious phylogeny, that can be erroneously incongruent to some extent with another phylogeny that has been correctly estimated [22,33,40]. Thus, given two data sets, one of which has parameters prone to long-branch attraction [46,47], the choice of an inconsistent phylogenetic method to analyse both data sets may produce different trees. Incongruence due to systematic errors can be addressed by changing the evolutionary model or the phylogenetic method so that it conforms better to the data. However, incongruence resulting from genealogical discordance processes must be detected and handled in some appropriate ways, e.g., by using phylogenetic network inference methods [see [48] for a review]. Thus, three main causes can be invoked to explain incongruence: 1) different phylogenetic trees may be inferred due to random sampling errors, 2) different trees may be produced due to the presence of systematic errors, leading to erroneous phylogenetic inference, or 3) real differences may exist between phylogenetic trees due to contrasting evolutionary histories [38].

Alternatively, the term "congruence" is often used to describe data sets, characters or trees that correspond to identical (or compatible) relationships among taxa [49]. However, many authors use a definition of congruence that is looser than the previously described *identical *topology and that incorporate varying degrees of topological similarities. For example, taxonomic congruence, as defined by [12], is the *degree *to which different classifications of the same taxa support the same groupings. Since the pioneer study of [12], different measures and indices have been proposed to quantify the level of congruence [see review by [18]]. Conditional data combination often relies on such indices to determine the degree of congruence and on statistical tests in order to determine whether or not the data sets should be combined [13,18,33].

As described above, the term "congruence" and "incongruence" can have a more or less strict meaning with regards to the level of similarity. The definitions used in this paper are in concordance with the test of congruence among distance matrices (CADM). CADM was introduced by [50] and is applicable to two or more matrices. The null hypothesis of the test (H_0_) is the complete incongruence of all trees (two or more), which corresponds to phylogenies with different topologies and/or very different branch lengths. Hence, the method can also account for branch lengths [as in [51]]. For two matrices (or two trees), the alternative hypothesis (H_1_) is that the inferred trees are partially or completely congruent. When more than two matrices (or trees) are tested, H_1 _postulates that *at least *two trees in the group are partially or completely congruent. It is then possible to test for specific pairs. In this paper, *incongruence *refers to phylogenetic trees with different topologies, which suggests completely distinct evolutionary histories. At the opposite, *congruence *refers to two or more identical trees with an underlying identical evolutionary history (i.e., *complete congruence *or topological identity) or to two or more phylogenetic trees with a partial degree of similarity in their evolutionary relationships (i.e., *partial congruence*). The level of congruence can be measured by the test statistic, which ranges from 0 to 1.

More specifically, given two or more data sets (e.g., different genes) studied on the same species, a concordance statistic [Kendall's *W *statistic: [52], [53]] is calculated among the distance matrices corresponding to the gene sequences or to the trees and tested against a distribution of permuted values to estimate the probability that the data correspond to the null hypothesis. CADM is an extension of the Mantel test of matrix correspondence, which can be used to test the null hypothesis of complete incongruence of the distance matrices (corresponding to all data sets or trees under study). As a complement to the p-value, the *W *statistic provides an estimate of the degree of congruence of two or more matrices on a scale between 0 (no congruence) and 1 (complete congruence). Note that when trees have identical topologies but different branch lengths, a statistical conclusion of partial congruence or even incongruence may be reached, depending on the level of differences among the distance matrices that cause differences in relative distance rankings. The test allows users to detect these two cases; thus, both topological and phylogenetic congruence can be tested with CADM (see the Methods section).

*A posteriori *tests can be used to identify which data sets are congruent and to estimate their level of congruence. When the level of congruence among all distance matrices is low, researchers can decide to analyse the matrices separately or in subgroups. However, rejection of independence of the gene trees does not imply that inference of a tree from the combined data set is appropriate. In the case of partial congruence, phylogenetic network methods [48,54], instead of traditional tree reconstruction methods, can be more appropriate to combine congruent data sets into a single analysis. Indeed, in these cases, evolutionary relationships may be better depicted as reticulated relationships. Additional tests or studies can also be performed to determine the causes underlying partial congruence [e.g., [55]]. Thus, with its null hypothesis (H_0_) of complete incongruence of all trees, CADM differs from most other available phylogenetic tests of congruence/incongruence, which assume a common evolutionary history (H_0_: congruence) and test the alternative hypothesis of different histories among the data sets (H_1_: incongruence).

Previously published simulations have shown that the global and *a posteriori *CADM tests have a correct type I error rate and good power when applied to dissimilarity matrices computed from independently-generated raw data [50]. Identical results were obtained in simulations involving ultrametric distance matrices [56]. CADM has also been successfully used to detect congruence among phylogenetic trees obtained from different gene sequences [57]. In this paper, we expand on previous CADM simulations to assess the performance of the test when it is applied to phylogenetic trees. Specifically, the type I error rate and power of the global and *a posteriori *CADM tests were assessed using distance matrices obtained from nucleotide sequences simulated on additive trees under various phylogenetic conditions.

## Results

### Type I error rate

Type I error rate was evaluated by calculating the proportion of replicated simulations that rejected the null hypothesis when H_0 _was true by construct. To construct data sets under a true H_0 _of complete incongruence among matrices, IM were compared using CADM. Table 1 presents type I error rates of the global CADM test, at a nominal significance level of 0.05, obtained for different numbers of IM (2, 3, 4, 5 and 10); n (10, 25, 50 and 100); and L (1000, 5000, 10 000 and 20 000 bp). In all cases, the 95% CI of the rejection rates included the nominal 0.05 alpha level, suggesting an adequate type I error rate when CADM is applied to compare distance matrices in a phylogenetic context. Type I error rates were also investigated for *a posteriori *CADM test, where matrices included in a set under comparisons are permuted one at a time. As for the global test, in all cases and for each matrix, the 95% CI included the nominal 0.05 alpha level, suggesting an adequate type I error rate (results not shown).

**Table 1:** Type I error rates for CADM simulations with nucleotide sequences matrices simulated on independently-generated additive trees under a GTR + Γ + I model of evolution.

		Number of IM				
		
n	L	2	3	4	5	10
10	1000	0.052	0.047	0.042	0.049	0.046
		(0.038, 0.066)	(0.034, 0.060)	(0.030, 0.054)	(0.036, 0.062)	(0.033, 0.059)
	5000	0.050	0.050	0.038	0.046	0.046
		(0.036, 0.064)	(0.036, 0.064)	(0.026, 0.050)	(0.033, 0.059)	(0.033, 0.059)
	10 000	0.049	0.048	0.046	0.046	0.047
		(0.036, 0.062)	(0.035, 0.061)	(0.033, 0.059)	(0.033, 0.059)	(0.034, 0.060)
	20 000	0.047	0.047	0.039	0.045	0.043
		(0.034, 0.060)	(0.034, 0.060)	(0.027, 0.051)	(0.032, 0.058)	(0.030, 0.056)

25	1000	0.054	0.056	0.054	0.056	0.04
		(0.040, 0.068)	(0.042, 0.070)	(0.040, 0.068)	(0.042, 0.070)	(0.028, 0.052)
	5000	0.053	0.048	0.046	0.05	0.042
		(0.039, 0.070)	(0.035, 0.061)	(0.033, 0.059)	(0.036, 0.064)	(0.030, 0.054)
	10 000	0.046	0.054	0.05	0.049	0.050
		(0.033, 0.059)	(0.040, 0.068)	(0.036, 0.064)	(0.036, 0.062)	(0.036, 0.064)
	20 000	0.043	0.050	0.054	0.047	0.040
		(0.030, 0.056)	(0.036, 0.064)	(0.040, 0.068)	(0.034, 0.060)	(0.028, 0.052)

50	1000	0.048	0.062	0.059	0.050	0.049
		(0.035, 0.061)	(0.047, 0.077)	(0.044, 0.074)	(0.036, 0.064)	(0.036, 0.062)
	5000	0.056	0.049	0.055	0.053	0.050*
		(0.042, 0.070)	(0.036, 0.062)	(0.041, 0.069)	(0.039, 0.070)	(0.007, 0.093)
	10 000	0.041	0.048	0.053	0.051	0.050*
		(0.029, 0.053)	(0.035, 0.061)	(0.039, 0.067)	(0.037, 0.065)	(0.007, 0.093)
	20 000	0.050	0.053	0.050	0.056	0.060*
		(0.036, 0.064)	(0.039, 0.067)	(0.036, 0.064)	(0.042, 0.070)	(0.012, 0.107)

100	1000	0.051	0.042	0.040	0.044	0.030*
		(0.037, 0.065)	(0.030, 0.054)	(0.028, 0.052)	(0.031, 0.057)	(-0.004,0.064)
	5000	0.066	0.040*	0.030*	0.050*	0.060*
		(0.051, 0.081)	(0.001, 0.079)	(-0.004,0.064)	(0.007, 0.093)	(0.013, 0.107)
	10 000	0.030*	0.060*	0.070*	0.050*	0.040*
		(-0.004,0.064)	(0.013, 0.107)	(0.019, 0.120)	(0.007, 0.093)	(0.001, 0.079)
	20 000	0.060*	0.050*	0.040*	0.060*	0.070*
		(0.013, 0.107)	(0.007, 0.093)	(0.001, 0.079)	(0.013, 0.107)	(0.019, 0.120)

Rejection rate are given at a significance level of 0.05, with 95% confidence intervals in parentheses. Calculated from 1000 replicates, except for cells with * (100 replicates). IM = incongruent matrix, n = number of taxa, L = nucleotide sequence length.

### Power: Different levels of congruence among matrices

**Figure 1:**
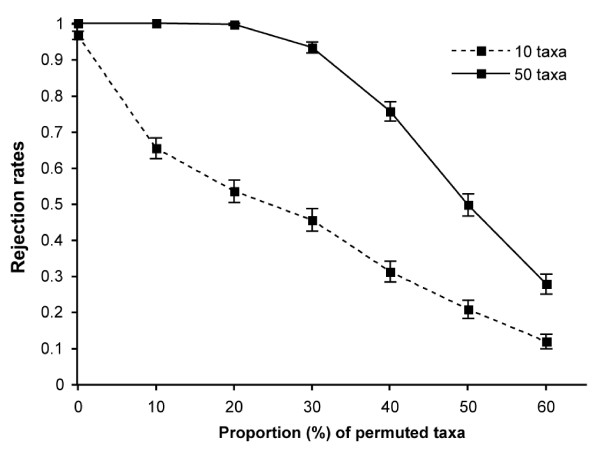
**Rejection rates of H_0 _for the *global *CADM test, comparing data sets simulated on partly similar trees, with identical evolutionary parameters (GTR+ Γ+ I)**. Three partially congruent matrices (CM**_P_**) and two incongruent matrices (IM) were included in each test, for a total of five distance matrices (M = 5). CM**_P _**were generated by permuting an increasing number of taxa from a total of 10 taxa (dashed line) and 50 taxa (solid line), which corresponds to different level of congruence, and for L = 10 000 bp. Rejection rates are given at a significance level of 0.05, with 95% confidence intervals represented by vertical lines, calculated from 1000 replicates.

The estimated power is the proportion of replicates for which the null hypothesis is rejected when H_0 _is false by construct. For 1000 replicates, a power of 1.0 (i.e., rejection rates of 1.0) indicates that all replicates rejected the false null hypothesis, and thus power is maximal. Figure 1 shows power curves obtained when different numbers of taxa were permuted to construct congruent matrices (CM = 3, M = 5), L = 10 000 bp and n = 10 and 50 taxa. When the proportion of permuted taxa is equal to 0, the distance matrices were obtained from nucleotide sequences simulated on identical trees (CM_I_). When the proportion of permuted taxa is greater than 0, the distance matrices were obtained from nucleotide sequences simulated on partly similar trees (CM_P_), and thus, it corresponds to different levels of partial congruence (depending on the number of permuted taxa). Power decreased with a decrease in the level of congruence among the three matrices (i.e., with an increase in the number of taxa permuted). A power close to 1.0 was observed when identical trees were used (CM_I_), regardless of matrix sizes (n). Reduced power (i.e., less than 0.5) was observed for matrices with 25% or more permuted taxa, when n = 10 taxa; whereas it was observed for matrices with 50% or more permuted taxa, when n = 50 taxa.

**Figure 2:**
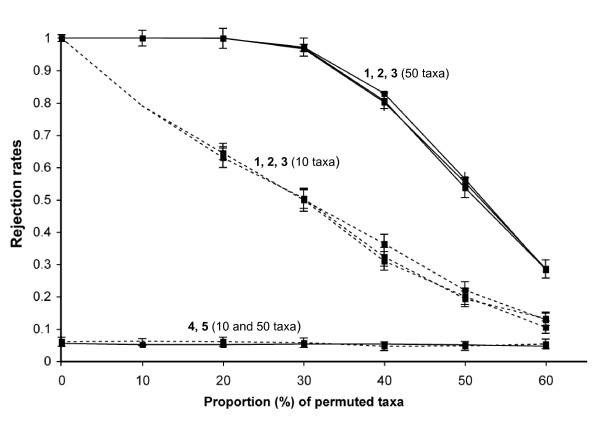
**Rejection rates of H_0 _for *a posteriori *CADM tests, comparing data sets simulated on partly similar trees, with identical evolutionary parameters (GTR+ Γ+ I)**. Three partially congruent matrices (CM**_P_**) and two incongruent matrices (IM) were included in each test, for a total of five distance matrices (M = 5). CM**_P _**were generated by permuting an increasing number of taxa from a total of 10 taxa (dashed line) and 50 taxa (solid line), which corresponds to different level of congruence, and for L = 10 000 bp. Rejection rates for the five distance matrices compared are given in Figure 1 for the global CADM test. For ***a posteriori ***tests, the power curves are given for each of the five matrices permuted separately, and numbered from 1 to 5. Rejection rates are given at a significance level of 0.05, with 95% confidence intervals represented by vertical lines, calculated from 1000 replicates.

In *a posteriori *CADM tests, the rejection rate of each individual matrix was similar to the power level obtained in the global test. Figure 2 presents the rejection rates obtained for each matrix tested individually for M = 5 (same matrices as those used for simulations presented in Figure 1). The three partly similar matrices (CM_P_) were obtained from nucleotide sequences simulated under identical evolutionary conditions. In situations where not all matrices are congruent in a replicate, incongruent matrices are expected to fail to reject the null hypothesis of incongruence (i.e., the rejection rate corresponding to type I error), whereas partially congruent matrices should reject H_0 _(i.e., the rejection rate corresponding to power). This is exemplified in Figure 2, where partially congruent matrices (matrices 1, 2 and 3) present rejection rates above 0.05, thus above the significance level alpha that was used for each test. Rejection rates for incongruent matrices 4 and 5 were near 0.05, for both n = 10 and 50 taxa. The power curves in Figure 2 are nearly identical to those in Figure 1 for data sets of the same size n, when partially congruent matrices were tested (matrices 1, 2 and 3). Tables 2 and 3 present rejection rates for M = 5, and that included a varying number of CM. In Table 2, CM correspond to distance matrices obtained from nucleotide sequences simulated on identical trees (CM_I_), whereas in Table 3, CM correspond to distance matrices obtained from nucleotide sequences simulated on partly similar trees with 40% of permuted taxa (CM_P_). For both tables, power increased with (1) an increase in the number of objects (n); (2) an increase in the number of congruent matrices (CM); and (3) an increase in sequence lengths (L). However, power increased much more rapidly in Table 2, which corresponds to a situation of *complete *congruence, with maximum power obtained when three or more CM were included in a replicate, regardless of matrix sizes (n). In Table 3, which corresponds to a situation of *partial *congruence, maximum power was observed only for four matrices or more, included in a set of M = 5, and with larger matrices (n = 100 taxa).

**Table 2:** Rejection rates of H_0 _for CADM comparing data sets simulated on *identical *trees and with *identical *evolutionary parameters (GTR+ Γ + I), M = 5.

		CM_I_			
**n**	**L**	**2**	**3**	**4**	**5**

10	1000	0.308	0.928	1.000	1.000
		(0.279-0.337)	(0.912-0.944)	-	-
	5000	0.363	0.973	1.000	1.000
		(0.333-0.393	(0.963-0.983)	-	-
	10 000	0.383	0.966	1.000	1.000
		(0.353-0.413)	(0.955-0.977)	-	-
	20 000	0.380	0.974	1.000	1.000
		(0.350-0.410)	(0.964-0.984)	-	-

25	1000	0.569	1.000	1.000	1.000
		(0.538-0.600)	-	-	-
	5000	0.662	1.000	1.000	1.000
		(0.633-0.691)	-	-	-
	10 000	0.675	1.000	1.000	1.000
		(0.646-0.704)	-	-	-
	20 000	0.682	1.000	1.000	1.000
		(0.653-0.711)	-	-	-

50	1000	0.740	1.000	1.000	1.000
		(0.715-0.769)	-	-	-
	5000	0.851	1.000	1.000	1.000
		(0.829-0.873)	-	-	-
	10 000	0.869	1.000	1.000	1.000
		(0.848-0.890)	-	-	-
	20 000	0.898	1.000	1.000	1.000
		(0.880-0.917)	-	-	-

100	1000	0.890*	1.000*	1.000*	1.000*
		(0.828-0.952)	-	-	-
	5000	0.970*	1.000*	1.000*	1.000*
		(0.936-1.000)	-	-	-
	10 000	0.970*	1.000*	1.000*	1.000*
		(0.936-1.000)	-	-	-
	20 000	0.970*	1.000*	1.000*	1.000*
		(0.936-1.000)	-	-	-

A false H_0 _was constructed by including a different number of completely congruent matrices (CM_I_) together with a different number of incongruent matrices (IM), for a total of five distance matrices (M = 5). When CM_I _= 5, all matrices included in the test are congruent. Rejection rates are given at a significance level of 0.05, with 95% confidence intervals in parentheses. Calculated from 1000 replicates, except for cells with * (100 replicates). Dashes (-) correspond to a CI of 1.000 - 1.000.

**Table 3:** Rejection rates of H_0 _for CADM comparing data sets simulated on *partly similar *trees and with *identical *evolutionary parameters (GTR+ Γ + I), M = 5.

		CM_P_			
**n**	**L**	**2**	**3**	**4**	**5**

10	1000	0.106	0.263	0.523	0.802
		(0.087-0.125)	(0.236-0.290)	(0.492-0.554)	(0.777-0.827)
	5000	0.105	0.300	0.586	0.866
		(0.086-0.124)	(0.272-0.328)	(0.555-0.617)	(0.845-0.887)
	10 000	0.113	0.311	0.608	0.872
		(0.093-0.133)	(0.282-0.340)	(0.578-0.638)	(0.851-0.893)
	20 000	0.122	0.314	0.615	0.875
		(0.102-0.142)	(0.285-0.343)	(0.585-0.645)	(0.854-0.896)

25	1000	0.130	0.409	0.805	0.977
		(0.109-0.151)	(0.378-0.440)	(0.780-0.830)	(0.968-0.986)
	5000	0.158	0.495	0.893	0.993
		(0.135-0.181)	(0.464-0.526)	(0.874-0.912)	(0.988-0.998)
	10 000	0.151	0.508	0.902	0.997
		(0.129-0.173)	(0.477-0.539)	(0.884-0.920)	(0.994-1.000)
	20 000	0.153	0.514	0.907	0.996
		(0.131-0.175)	(0.483-0.545)	(0.889-0.925)	(0.992-1.000)

50	1000	0.163	0.560	0.960	1.000
		(0.140-0.186)	(0.529-0.591)	(0.948-0.972)	-
	5000	0.206	0.701	0.991	1.000
		(0.181-0.231)	(0.673-0.729)	(0.985-1.000)	-
	10 000	0.218	0.730	0.996	1.000
		(0.192-0.244)	(0.702-0.758)	(0.992-1.000)	-
	20 000	0.229	0.748	0.997	1.000
		(0.203-0.255)	(0.721-0.775)	(0.994-1.000)	-

100	1000	0.210*	0.730*	0.990*	1.000*
		(0.129-0.291)	(0.641-0.819)	(0.970-1.000)	-
	5000	0.260*	0.880*	1.000*	1.000*
		(0.173-0.347)	(0.815-0.945)	-	-
	10 000	0.270*	0.900*	1.000*	1.000*
		(0.181-0.359)	(0.840-0.960)	-	-
	20 000	0.310*	0.920*	1.000*	1.000*
		(0.218-0.402)	(0.866-0.974)	-	-

A different number of partially congruent matrices (CM_P_) and a different number of incongruent matrices (IM) were included in each test, for a total of five distance matrices (M = 5). To generate CM_P_, nucleotide sequences were simulated on partly similar trees (with permutations of 40% of n). Rejection rates are given at a significance level of 0.05, with 95% confidence intervals in parentheses. Calculated from 1000 replicates, except for cells with * (100 replicates). Dashes (-) correspond to a CI of 1.000 - 1.000.

### Power: Effect of different evolutionary parameters

Power was also calculated for distance matrices obtained from nucleotide sequences simulated on identical trees under a GTR model, with identical or different evolutionary parameters. In Table 4, distance matrices obtained from nucleotide sequences simulated with *identical *evolutionary parameters were compared, for CM_I _= 2. Rejection rates of pairwise comparisons tested for different values of mutation rates (s) and heterogeneity of substitution rates among sites (α) are presented. In Table 5, distance matrices obtained from nucleotide sequences simulated with *contrasting *evolutionary parameters were compared. In both cases, and for every condition tested, rejection rates were high, with at least 79% of the replicates rejecting H_0_. The lowest rejection rate was observed when matrices obtained from nucleotide sequences simulated under more extreme parameters of evolution (i.e., s = 0.02; α = 0.06) were tested. But in general, most cases rejected the null hypothesis of complete incongruence (i.e., power of 1.0). Identical simulations were also performed for CM_I _= 5, M = 5; and rejection rates were of 1.0 in every case (results not shown).

**Table 4:** Rejection rates of H_0 _for CADM comparing data sets simulated on *identical *trees (CM_I _= 2, M = 2) and with *identical *evolutionary parameters.

		α = 0.06		α = 0.8168		α = 200	
n	L	s = 0.02	s = 0.4	s = 0.02	s = 0.4	s = 0.02	s = 0.4
10	1000	0.789	0.958	0.944	1.000	0.940	1.000
		(0.764-0.814)	(0.946-0.970)	(0.930-0.958)	-	(0.925-0.955)	-
	5000	0.999	1.000	1.000	1.000	1.000	1.000
		(0.997-1.000)	-	-	-	-	-
	10 000	1.000	1.000	1.000	1.000	1.000	1.000
		-	-	-	-	-	-
	20 000	1.000	1.000	1.000	1.000	1.000	1.000
		-	-	-	-	-	-

50	1000	0.891	0.997	0.976	1.000	0.978	1.000
		(0.872-0.910)	(0.994-1.000)	(0.966-0.986)	-	(0.969-0.987)	-
	5000	1.000	1.000	1.000	1.000	1.000	1.000
		-	-	-	-	-	-
	10 000	1.000	1.000	1.000	1.000	1.000	1.000
		-	-	-	-	-	-
	20 000	1.000	1.000	1.000	1.000	1.000	1.000
		-	-	-	-	-	-

Results are shown for a GTR + Γ + I model with different s and α. Rejection rates are given at a significance level of 0.05, with 95% confidence intervals in parentheses. Calculated from 1000 replicates.

**Table 5:** Rejection rates of H_0 _for CADM comparing data sets simulated on *identical *trees (CM_I _= 2, M = 2), with *different *evolutionary parameters (GTR model with different s or α, for each data set).

		s = 0.02		s = 0.4		α = 0.06	α = 0.8168	α = 200
		
n	L	α: 200 vs. 0.06	α: 200 vs. 0.8168	α: 200 vs. 0.06	α: 200 vs. 0.8168	s: 0.02 vs. 0.4	s: 0.02 vs. 0.4	s: 0.02 vs. 0.4
10	1000	0.866	0.939	0.993	1.000	0.949	0.998	0.999
		(0.845-0.887)	(0.924-0.954)	(0.988-0.998)	-	(0.935-0.963)	(0.995-1.000)	(0.997-1.000)
	5000	1.000	1.000	1.000	1.000	1.000	1.000	1.000
		-	-	-	-	-	-	-
	10 000	1.000	1.000	1.000	1.000	1.000	1.000	1.000
		-	-	-	-	-	-	-
	20 000	1.000	1.000	1.000	1.000	1.000	1.000	1.000
		-	-	-	-	-	-	-

25	1000	0.927	0.965	1.000	1.000	0.992	1.000	1.000
		(0.911-0.943)	(0.954-0.976)	-	-	(0.986-0.998)	-	-
	5000	1.000	1.000	1.000	1.000	1.000	1.000	1.000
		-	-	-	-	-	-	-
	10 000	1.000	1.000	1.000	1.000	1.000	1.000	1.000
		-	-	-	-	-	-	-
	20 000	1.000	1.000	1.000	1.000	1.000	1.000	1.000
		-	-	-	-	-	-	-

50	1000	0.945	0.980	1.000	1.000	0.999	1.000	1.000
		(0.931-0.959)	(0.971-0.989)	-	-	(0.997-1.000)	-	-
	5000	1.000	1.000	1.000	1.000	1.000	1.000	1.000
		-	-	-	-	-	-	-
	10 000	1.000	1.000	1.000	1.000	1.000	1.000	1.000
		-	-	-	-	-	-	-
	20 000	1.000	1.000	1.000	1.000	1.000	1.000	1.000
		-	-	-	-	-	-	-

Rejection rates are given at a significance level of 0.05, with 95% confidence intervals in parentheses. Calculated from 1000 replicates.

## Discussion

Incongruence among data sets is widespread in phylogenetic analyses [58]. Our simulations clearly demonstrate the validity of CADM to estimate the level of congruence (or to detect incongruence) among different data sets or partitions and to test its statistical significance. However, in comparison to other tests used in phylogenetic analysis, the null and alternative hypotheses are reversed. Most phylogenetic incongruence tests assume that the data sets share an identical evolutionary history and the null hypothesis is congruence (i.e., topological identity) among trees [38]. On the contrary, the null hypothesis of CADM is complete incongruence. Kendall's coefficient of concordance *W *is widely used in other fields, especially psychology, where it is used to assess the degree of correspondence or strength of association among different estimators [53,59]. In a phylogenetic context, CADM evaluates the level of congruence, i.e., the degree of agreement among different estimators (data partitions or genes) of phylogenies, represented by their evolutionary distances, and can be used to test for congruence among matrices or trees.

In order to investigate type I error rates, which is the proportion of replicates that rejected H_0 _when it was true by construct, incongruent distance matrices were compared. In every case, the 95% CI of the rejection rate included the nominal significance level of 0.05 used for the test (Table 1). Hence, CADM accurately detects completely incongruent matrices even when multiple data partitions are tested simultaneously. In comparison, the incongruence length difference test [ILD, [39]] produces inflated type I error rates under particular conditions. Computer simulations were designed in [41] to assess the performance of ILD under different conditions of rejection of the null hypothesis (i.e., congruence between data sets). In that paper, observed rejection rates were well above the alpha level when sequences were simulated on identical trees, but with important differences in the substitution rates among sites. Furthermore, the rejection rates increased for longer sequences and for asymmetrical trees. Similarly, ILD was shown to be strongly biased in detecting topological congruence [40], and to be negatively influenced by the presence of a substantial number of noisy characters [60]. Different methods have been proposed to alleviate this problem, such as using an alternative null model [60] or an arcsine transformation of the standardized length of the trees in order to linearism the relationship between noise and tree length [61].

Numerous congruence tests have also been designed recently such as principal component analysis on log-likelihood ratios or p-values [62], or heat maps to identify groups of congruent markers [63,64]. Bayesian approaches have also been suggested [65,66]. Caveats associated to each method are discussed in [67], where a hierarchical clustering method based on log-likelihood ratios [35] is introduced to test congruence. However, these tests are dependent upon tree inference [67], and thus could produce spurious results if inadequate models of evolution are used [68,69]. When using the CADM procedure, it is possible to compare the results obtained by computing a distance matrix on the nucleotide sequences using different distances or different substitution models to investigate possible bias. Alternatively, CADM can also be applied to the path-length distance matrices obtained from phylogenetic trees [57]. These different possibilities were all tested in preliminary simulations (not reported here), and no effect on the outcome of the test was observed.

As observed in previous simulation studies [50,56], the power of CADM increased with the number of taxa, with the level of congruence and with the number of congruent matrices within a set of distance matrices (Figures 1 and 2, Tables 2 and 3). Thus, the test performs according to expectations. Indeed, the power of a test should increase with the number of objects and with effect size, that is, the degree to which congruence is present [70]. Interestingly, power also tends to increase with longer nucleotide sequences from which distance matrices are calculated. This novel observation is opposed to the prediction in [50], where it is showed that power is not affected by the number of variables in the raw data. These authors argue that the number of variables should not affect the outcome of the test since data partitions are converted into distance matrices prior to computing the test. However, a weighted version of CADM, which can be used to assign weights to each matrix in the global analysis, is presented in [50]. Comparison of distance matrices obtained from nucleotide sequences is a particular application of the CADM test. We believe that the higher power observed for longer sequences can be explained by the number of informative sites. However, it appears that power increases more rapidly with the number of taxa than with the number of characters, and even more rapidly with the number of congruent matrices under comparison.

When the overall level of congruence decreases among congruent matrices, so does power (Figure 1). For nucleotide sequence matrices simulated on phylogenetic trees with 40% permuted taxa (partial congruence), a drastic decrease in power was observed when compared to nucleotide sequence matrices simulated on identical phylogenetic trees (complete congruence, Table 2 vs. Table 3). The greater the effect size, the greater the power of the test will be [70]. Tables 4 and 5 present the rejection rates for two matrices that have been simulated on identical trees (i.e., complete congruence), and nearly all cases tested rejected the null hypothesis of complete incongruence, regardless of the evolutionary parameters used. In this study, topological differences are reflected by a decrease in congruence among the sequence matrices, and this can be interpreted as noise in the data or to real incongruence. Indeed, power decreases quite abruptly with an increase in topological differences (Figure 1). The level of congruence among distance matrices is indicated by the statistic value in *a posteriori *tests.

One of the main advantages of CADM lies in its ability to test several matrices in a single analysis, and identify partially or completely congruent and incongruent members of a set of matrices. This is achieved through *a posteriori *testing, which compares each matrix to all other matrices by permuting a single matrix at a time. Our results show that power of *a posteriori *CADM tests is equivalent to the power observed for the global test (Figures 1 and 2). When the null hypothesis of incongruence is rejected, *a posteriori *tests should be used to identify the matrices that can be combined in a supermatrix, and those that should be analysed separately, or combined in a supernetwork approach [48,54]. Other tests, such as ILD, can be modified to test for incongruence among multiple matrices. Different approaches have been proposed to identify incongruent matrices within a set of multiple matrices; the methods and problems associated are discussed in [38,71]. The CONCATERPILLAR program also allows testing for incongruence among multiple matrices through pairwise comparisons [67]. However, the number of tests increases exponentially with the number of data sets, and it becomes excessively computationally demanding when numerous data sets have to be compared.

## Conclusions

In the light of our results, CADM has proven to be statistically valid to detect partial or complete congruence among distance matrices and estimate its level in a phylogenetic context. One important advantage of this permutation method is its computational efficiency in significance testing. CADM offers several other advantages with respect to previously described incongruence tests: (1) The statistic is calculated directly from the distance matrices, thus different types of data can be compared after convertion to distance matrices using an appropriate function. (2) Data that readily come in the form of distance matrices do not have to be further transformed into character-state data matrices. (3) Given that distances can be calculated directly from the raw data without inferring a phylogenetic tree, possible biases introduced by the use of an inappropriate phylogenetic method can be reduced. (4) Also, appropriate distances can be chosen for each individual data set to accurately model its evolutionary parameters. (5) If needed, path-length distances calculated on phylogenetic trees can also be used, which provide an interesting method to test for congruence among different trees in a supertree approach. (6) Distance matrices can be weighted differentially to account for different numbers of characters. (7) *A posteriori *tests can be performed to identify which particular matrices are congruent among all data sets tested. (8) An estimate of the level of congruence can be obtained by the statistic value of *a posteriori *test. With the growing amount of taxa and sequences that are used in phylogenomics, CADM offers a simple alternative to compare multiple matrices and identify congruent data partitions. CADM could also be used in cophylogeny studies, where congruence between species phylogenies is assessed to determine the level of host specificity [e.g., [72-74]]. Further simulations designed specifically in a coevolution, cospeciation or cophylogeny context could be performed to validate the use of CADM in these particular settings.

## Methods

### CADM test

**Figure 3:**
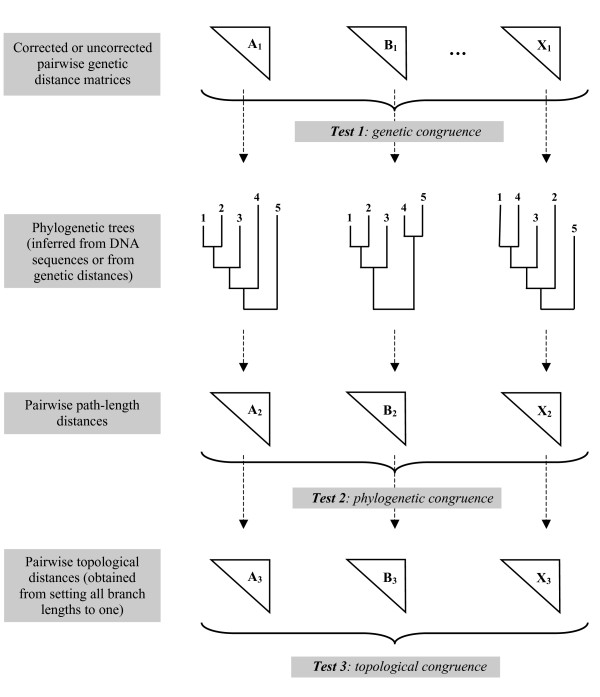
**Performing tests of incongruence**. Three different incongruence tests are possible: directly on the pairwise genetic distance matrices (Test 1), on the path-length distance matrices corresponding to the phylogenetic trees (Test 2), and on the topological distance matrices obtained by setting all branch length to 1 in the phylogenies (Test 3).

**Figure 4:**
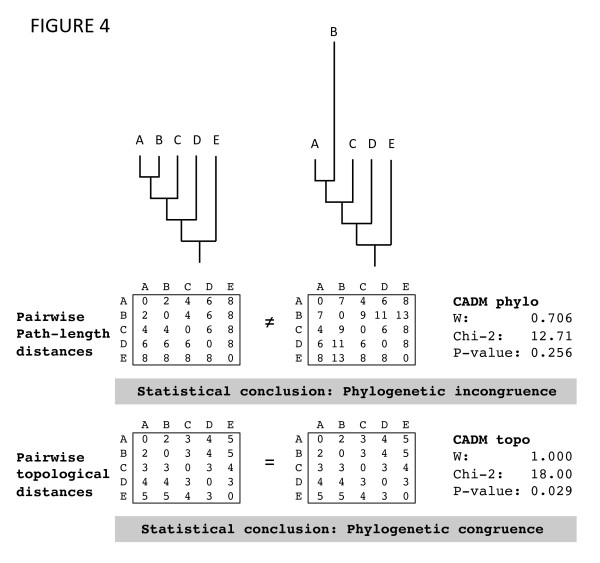
**Application of the CADM test**. Graphical and numerical example showing a particular case for which two phylogenetic trees are incongruent in their path-length distances (Test 2, Figure 3) but topologically congruent (Test 3, Figure 3).

The null hypothesis (H_0_) of the global CADM test is the complete incongruence of the matrices under study, whether these matrices contain pairwise genetic distances, pairwise path-length distances, or pairwise topological distances. Rejecting H_0 _indicates that at least two matrices contain a certain amount of congruent information. The global statistic value measures the level of congruence for partially congruent matrices, with a maximum value of 1 indicating complete congruence among the matrices (i.e., identical rankings of distance matrices). One advantage of the test is that congruence can easily be detected and measured at different steps of the analysis, since CADM can be applied to any type of distance matrices (Figure 3). Thus, it is possible to distinguish between different types of congruence: (1) genetic congruence, (2) phylogenetic congruence, and (3) topological congruence. Genetic congruence can be tested through comparisons of distance matrices calculated on the sequence data (with an uncorrected measure, or one corrected with an evolutionary model, see Test 1: Figure 3). Alternatively, the test can be applied to the path-length distance matrices corresponding to the inferred trees in order to determine the overall phylogenetic congruence (see Test 2: Figure 3). Topologically congruent trees with branch lengths that differ enough to change the ranking of the values may be considered incongruent (see the example given in Figure 4). To detect such cases, an additional test can be performed by setting all branch lengths to 1, so that only topologies are represented by the distance matrices (see Test 3: Figure 3). Preliminary simulations showed that the CADM test had an adequate type I error rate and good power, whether it is applied to genetic or phylogenetic distance matrices. Thus, most of the simulations were performed on genetic distance matrices because this approach allows users to determine, before phylogenetic inference, if the data matrices must be treated in a separate or combined analysis.

*A posteriori *tests can be used to identify incongruent and congruent matrices in a set. To determine the groups of potentially congruent matrices that could be tested, matrix correlations (also called Mantel statistics) based on ranks can be used. The test statistic *W *gives an estimate of the level of congruence, which ranges from 0 (complete incongruence) to 1 (complete congruence). After CADM tests, the completely congruent matrices can be combined in a supermatrix analysis, or analysed together using a supernetwork approach in the case of partially congruent matrices [e.g., [54], [75]]. A summary of the computations to perform the CADM test follows:

• The upper off-diagonal section of each distance matrix is unfolded and written into a vector corresponding to row *i *in a worktable.

• The entries of each row are transformed into ranks according to their values.

• The sum of ranks (*R_j_*) is calculated for each column *j *of the table.

• The mean () of all *R_j _*values is calculated.

• The Kendall coefficient of concordance (*W*) is computed using the following formula:

where *p *is the number of matrices, *n *is the number of distances in each matrix, *S *is obtained using:

and *T *is a correction factor for tied ranks:

in which *t_k _*is the number of tied ranks for each *k *of *m *groups of ties. Kendall's *W *statistic is simply the variance of the row sums of ranks *R_j _*divided by the maximum possible value that this variance can take, which occurs when all data matrices are in total agreement. Thus, *W *ranges from 0 to 1, where 0 represents a complete disagreement in the rankings of the distances among the different matrices, and a value of 1 is observed when the distance matrices are in complete agreement.

*• W *is transformed into a Friedman's *χ*^2^, which is a pivotal statistic appropriate for testing, using the following formula:

• The observed Friedman's *χ*^2 ^() is tested against a distribution of the statistic obtained under permutation (*χ*^2^*); the true value, , is included in the distribution of the permuted values, *χ*^2^*. For the global CADM test, all matrices are permuted at random, whereas for *a posteriori *tests, each matrix is permuted alternatively. A matrix that is not congruent to any other will have a small impact on the statistic once permuted. After a number of permutations (*p_n_*), the one-tailed probability of the data under H_0 _is computed as the number of χ^2^* values greater than or equal to  divided by (*p_n _*-1). In *a posteriori *comparisons, the p-value should be adjusted to maintain an adequate experimentwise error rate using a method designed specifically to correct for multiple testing. More details about the CADM procedure can be found in [50] and [56]. A version of CADM is available in R 2.9.0 [76,77], within the Ape 2.3 package [78,79].

For the simulations described below, one thousand replicates were simulated for each combination of parameters, unless stated otherwise. For each replicate, 999 random permutations were computed to estimate the reference distribution of the CADM statistic. We calculated the rate of rejection of H_0 _with its 95% confidence interval (CI), at a nominal significance level of 0.05, for cases where H_0 _was true (type I error rate) and for cases where H_0 _was false (power). All the analyses were performed on ten Power Mac G5, with PowerPC 970 MP processors (2 × 2.5 GHz).

### Type I error rate

**Figure 5:**
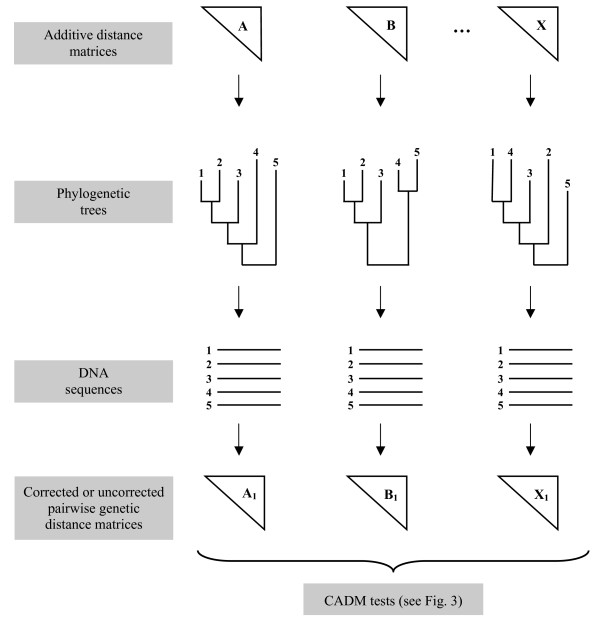
**Simulation protocol to generate distance matrices**. The simulation protocol involves three steps: 1) additive distance matrices (A to X) are generated, 2) phylogenetic trees are inferred, and 3) DNA sequences are simulated on the trees.

The type I error rate, which is the probability of rejecting H_0 _when the data conform to this hypothesis, was assessed for both the global and *a posteriori *CADM tests. A statistical test is valid if the rejection rate of H_0 _is smaller than or equal to the nominal significance level of the test [80]. Given that H_0 _postulates complete incongruence resulting from independent phylogenetic processes, we considered H_0 _to be true by construct when distance matrices calculated on nucleotide sequences simulated on independently-generated phylogenetic trees were compared. To do so, random additive distance matrices were obtained using the method proposed by [51]. Such distance matrices correspond to trees with random topologies, random permutations of the taxon labels, and branch lengths assigned at random from the distribution of actual branch lengths. Phylogenetic trees were computed from the distance matrices using a neighbor joining algorithm [NJ: [81]] in PAUP* 4.0 [82]. Nucleotide sequences were simulated on the phylogenetic trees using Seq-Gen 1.3.2 [83]. To reproduce the complexity of actual substitutions observed in real sequence data, we used a general time-reversible model [GTR: [84-86]] following a gamma distribution [Γ: [87]] with invariant sites (I). Parameters were identical to those used in [88]. Accordingly, the equilibrium frequencies of nucleotides A, C, G, and T were: gA = 0.1776, gC = 0.3336, gG = 0.2595, gT = 0.2293, the relative substitution rates were: rAC = 3.297, rAG = 12.55, rAT = 1.167, rCG = 2.060, rCT = 13.01, rGT = 1.0, and parameters α and I were 0.8168 and 0.5447 respectively. Distance matrices were calculated from the nucleotide sequence matrices using a *p *distance [89], corrected with the same parameters as those used to simulate the sequences. Given that the sequences were simulated on randomly-generated phylogenetic trees, the distance matrices obtained are incongruent matrices (IM). In order to explore various situations that might be encountered in phylogenetic analysis, different conditions were tested: different number of independent distance matrices (IM = 2, 3, 4, 5 and 10), different number of taxa in each matrix (n = 10, 25, 50 and 100) and varying lengths of nucleotide sequences (L = 1000, 5000, 10 000 and 20 000 bp). The simulation protocol is illustrated in Figure 5.

### Power

Power, which is the rate of rejection of a false H_0_, was evaluated for different conditions of application of CADM. Rejection rates of H_0 _were calculated with sets of distance matrices that included varying numbers of congruent matrices (CM) with different levels of similarity and different evolutionary parameters. The number of matrices (M) varied in a set and included incongruent matrices (IM) in addition to CM, for cases where CM < M.

### Power: Different levels of congruence among matrices

Nucleotide sequences were simulated under a GTR + Γ + I model on the NJ trees obtained from partly similar matrices (CM_P_) and from identical matrices (CM_I_). CM_P _were generated by random permutations of different numbers of taxa and branch lengths from a random additive distance matrix. As the number of permuted taxa increases, so does the distortion of the original matrix, whereas the level of congruence among matrices decreases. The number of taxa permuted varied according to the total number of taxa (n) included in each matrix, in order to maintain the same proportion of the taxa permuted regardless of the matrix size. The effect of the level of congruence on power was tested for CM_P _= 3, out of a total of five matrices (M = 5), with n = 10 or 50, and L = 10 000 bp. The power of *a posteriori *tests was also investigated with the same sets of CM_P_. The number of taxa permuted varied from 0 to 60% of the total number of taxa. Additional simulations were performed to compare the particular case of 0% permuted taxa, which correspond to CM_I _(i.e., near 100% congruence among matrices) to CM_P _with 40% permuted taxa. For these analyses, a total of five distance matrices were compared (M = 5) but with varying number of CM_I _or CM_P _(i.e., 0, 2, 3, 4 or 5); n = 10, 25, 50 or 100; and L = 1000, 5000, 10 000 or 20 000 bp. When CM_I _or CM_P _= 0, only incongruent matrices (IM) were included in the set of five matrices, which corresponds to a true H_0_. A false H_0 _was constructed when CM_I _or CM_P _≥ 2, and all matrices were congruent when CM_I _or CM_P _= 5.

### Power: Effect of different evolutionary parameters

Because genes controlled by different evolutionary processes can share an identical evolutionary history (i.e., branching pattern), we investigated the effect of different evolutionary parameters on the power of the CADM test. Following [58], nucleotide sequences were simulated under the GTR + Γ + I model described above but with different mutation rates (s = 0.02 and 0.4) and different heterogeneity levels of substitution rates among sites (α = 0.06 and 0.8168). Homogeneity of substitution rates among sites were simulated using α = 200. The same phylogenetic tree was used to simulate nucleotide sequence matrices representing different partitions within a replicate, but different tree topologies were used for each replicate. Nucleotide sequence matrices simulated with *identical *or *different *evolutionary parameters on an identical tree were compared for M = 2 or 5; s = 0.02 or 0.4; α = 0.06, 0.8168 or 200; CM_I _= 2 or 5; n = 10, 25, 50 or 100; and L = 1000, 5000, 10 000 or 20 000 bp. Nucleotide sequences were simulated under the same GTR parameters as above, except for s and α that varied. Thus, in addition to comparing data sets that evolved under identical conditions, we also compared data sets that were simulated with different s or α values. Thus, for s = 0.02 and 0.4, we compared data sets characterized by heterogeneity of substitution among sites vs. data sets with a homogeneous substitution rate (α = 0.06 vs. α = 200, and α = 0.8168 vs. α = 200); and for α = 0.06, 0.8168 and 200, we compared data sets with a low mutation rate vs. a high mutation rate (s = 0.02 vs. s = 0.4).

## Authors' contributions

FJL and VC conceived the simulation protocol and participated in its design and realization. PL originally conceived and programmed the CADM method. For this paper, he participated in the elaboration of the simulation protocol and wrote R-language functions for CADM. VC performed the simulations and wrote the manuscript. All authors read, commented, and approved the final manuscript.
